# Enterovirus D68 Surveillance, St. Louis, Missouri, USA, 2016

**DOI:** 10.3201/eid2411.180397

**Published:** 2018-11

**Authors:** Mythili Srinivasan, Angela Niesen, Gregory A. Storch

**Affiliations:** Washington University, St. Louis, Missouri, USA (M. Srinivasan, G. Storch);; St. Louis Children’s Hospital, St. Louis (A. Niesen)

**Keywords:** surveillance, enterovirus D68, viruses, St. Louis, Missouri, USA

## Abstract

A fall 2016 outbreak of enterovirus D68 infection in St. Louis, Missouri, USA, had less effect than a fall 2014 outbreak on hospital census, intensive care unit census, and hospitalization for a diagnosis of respiratory illness. Without ongoing surveillance and specific testing, these cases might have been missed.

The largest known outbreak of enterovirus D68 (EV-D68) occurred in the United States in 2014 (*1*). Severe respiratory illnesses increased in fall of 2014, corresponding to a period when EV-D68 was present in the community, at St. Louis Children’s Hospital (St. Louis, Missouri, USA) and elsewhere in the United States (*1**,**2*). Multiple reports suggested that the predominant virus was from clade B1, although some viruses from clades B2 were also detected (*3**–**5*). During 2015, there were few reports of EV-D68 circulating in the United States (*6*); however, in 2016, EV-D68 reappeared in multiple US locations (New York, Colorado); virus sequences suggested that the predominant virus was from clade B3 (*4*,*7*). We also documented EV-D68 activity in St. Louis in 2016. Sequencing of viruses from 2 patients tested in the St. Louis Children’s Hospital virology laboratory revealed clade B3 with 99% identity to the clade B3 virus from New York (*8*). Our goal with this study was to determine if the 2016 outbreak had caused an increase in hospital census or increase in patients admitted with respiratory diagnosis, as was seen during the 2014 outbreak.

During August 7, 2016, through December 16, 2016, we used a previously described EV-D68–specific PCR to test 5%–10% of enterovirus/rhinovirus–positive samples submitted each week to the St. Louis Children’s Hospital diagnostic virology laboratory. The samples had been obtained from patients seen at the hospital’s emergency department or clinics or admitted to the inpatient units and had been routinely tested by a FilmArray Respiratory Panel (BioFire, Salt Lake City, UT, USA) (*9*). Samples were selected by laboratory staff without regard to patient characteristics and were deidentified before EV-D68 testing. We obtained inpatient and intensive care unit (ICU) census data for all patients (not limited to those with a respiratory diagnosis) and discharge diagnoses for hospitalized patients from the hospital’s Health Information Management System, an administrative database, for 2013–2016. 

Discharge diagnoses were categorized as respiratory or nonrespiratory. A respiratory diagnosis was defined as any principal diagnosis referring to disease processes of the respiratory tract (e.g., asthma exacerbation, bronchiolitis, respiratory distress, respiratory failure, pneumonia). We used the Pearson χ^2^ test of independence to compare the frequency distribution of patients with a respiratory diagnosis in 2014 and 2016 with frequency of those in 2013 and 2015 combined. All analyses were done with SAS/STAT software version 9.4 for Windows (SAS Institute, Cary, NC, USA). The Washington University Institutional Review Board determined that this project did not meet the definition of human subject research and, as such, was not subject to institutional review board review.

During August 7–November 5, 2016, we tested a total of 4,190 samples by using the viral respiratory panel; 1,058 (25%) were positive for rhinovirus/enterovirus. Further testing of 179 samples positive for rhinovirus/enterovirus revealed that 19 (11%) were positive for EV-D68 (Technical Appendix Figure, panel A). During November 6–26, 2016, we tested 47 rhinovirus/enterovirus–positive samples, and 4/47 (9%) were positive for EV-D68. During November 27–December 10, 2016, we tested 33 rhinovirus/enterovirus–positive samples, and none were positive for EV-D68. We did not include EV-D68 testing data from November 6–December 10, 2016, in the Technical Appendix Figure because of the difficulty in obtaining discharge diagnosis data (changes in registration systems affected data collection during that period). 

In contrast to the experience in 2014, overall inpatient or ICU census did not increase during this outbreak except for a 1–2 week period in October (Technical Appendix Figure, panels B–D). During August 7–November 5, 2014, the number and percentage of patients hospitalized with a respiratory diagnosis increased significantly (852/5,894, 14%) compared with the corresponding periods in 2013 and 2015 combined (1,156/10,958, 11%; p<0.0001). This increase in patients hospitalized with respiratory diagnoses in 2014 overlapped with the increase in EV-D68 activity in our hospital. In 2014, we tested 572 rhinovirus/enterovirus–positive specimens from August 3, 2104–October 31, 2014, and 159 (28%) were positive for EV-D68 (*2*). In contrast, the number and percentage of patients admitted with a respiratory diagnosis during August 7–November 5, 2016, decreased significantly (483/5,304, 9%) compared with the corresponding periods in 2013 and 2015 combined (p = 0.004).

The overall effects of the 2016 outbreak seem to have been less than those of the 2014 outbreak. The epidemiologic data from our hospital, which has a broad catchment area in the central United States, confirm that the period of EV-D68 activity in 2016 had less effect on hospital census, ICU census, and hospitalization for respiratory diagnosis than that in 2014. Although the measured parameters are relatively crude, we found no changes in data collection procedures that explain the observed differences, suggesting that the differences are the result of lower levels of EV-D68 circulation in the population in 2016. Our study suggests that surveillance using specific testing is needed to detect EV-D68 activity, which would have been missed if we had monitored only for increases in patients with respiratory diagnoses or hospital census.

Technical Appendix Numbers of samples tested for enterovirus D68, number of patients with respiratory diagnosis, and inpatient and intensive care unit daily census for patients admitted for any illness to St. Louis Children’s Hospital, St. Louis, Missouri, USA, 2013–2016.
