# Supplementary material for: Enterovirus D68 Surveillance, St. Louis, Missouri, USA, 2016
Source: Emerg Infect Dis. 2018 Nov;24(11):2115–7. doi: 10.3201/eid2411.180397 (PMC6199992; doi:10.3201/eid2411.180397)
Supplement: Technical Appendix — Numbers of samples tested for enterovirus D68, number of patients with respiratory diagnosis, and inpatient and intensive care unit daily census for patients admitted for any illness to St. Louis Children’s Hospital, St. Louis, Missouri, USA, 2013–2016. [file 18-0397-Techapp-s1.pdf]

Article DOI: https://doi.org/10.3201/eid2411.180397

Enterovirus D68 Surveillance, St. Louis, Missouri, USA, 2016

Technical Appendix

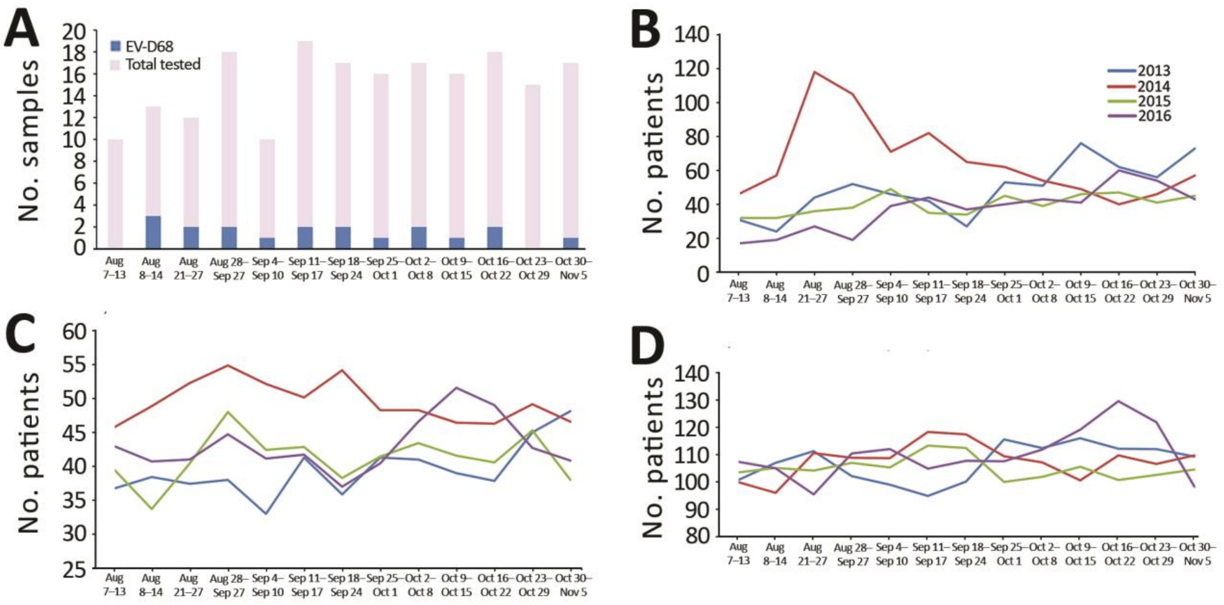

Technical Appendix Figure. Numbers of samples tested for enterovirus D68 (A), number of patients with respiratory diagnosis (B), and inpatient (C) and intensive care unit (D) daily census for patients admitted for any illness to St. Louis Children's Hospital, St. Louis, Missouri, USA, 2013–2016. Discharge diagnoses were categorized as respiratory or nonrespiratory. Respiratory diagnoses were defined as any principal diagnosis referring to disease process of the respiratory tract.
